# Galectin-1 as an Emerging Mediator of Cardiovascular Inflammation: Mechanisms and Therapeutic Opportunities

**DOI:** 10.1155/2018/8696543

**Published:** 2018-11-05

**Authors:** Ignacio M. Seropian, Germán E. González, Sebastián M. Maller, Daniel H. Berrocal, Antonio Abbate, Gabriel A. Rabinovich

**Affiliations:** ^1^Servicio de Hemodinamia y Cardiología Intervencionista, Instituto de Medicina Cardiovascular, Hospital Italiano de Buenos Aires, Buenos Aires C1199, Argentina; ^2^Instituto de Biología y Medicina Molecular, Consejo Nacional de Investigaciones Científicas y Técnicas (CONICET), Instituto de Fisiopatología Cardiovascular, Departamento de Patología, Universidad de Buenos Aires, Buenos Aires C1428, Argentina; ^3^Laboratorio de Inmunopatología, Instituto de Biología y Medicina Experimental (IBYME), Consejo Nacional de Investigaciones Científicas y Técnicas (CONICET), Buenos Aires C1428, Argentina; ^4^Pauley Heart Center, Virginia Commonwealth University, Richmond, VA 23298, USA; ^5^Departamento de Química Biológica, Facultad de Ciencias Exactas y Naturales, Universidad de Buenos Aires, Buenos Aires C1428, Argentina

## Abstract

Galectin-1 (Gal-1), an evolutionarily conserved *β*-galactoside-binding lectin, controls immune cell homeostasis and tempers acute and chronic inflammation by blunting proinflammatory cytokine synthesis, engaging T-cell apoptotic programs, promoting expansion of T regulatory (Treg) cells, and deactivating antigen-presenting cells. In addition, this lectin promotes angiogenesis by co-opting the vascular endothelial growth factor receptor (VEGFR) 2 signaling pathway. Since a coordinated network of immunomodulatory and proangiogenic mediators controls cardiac homeostasis, this lectin has been proposed to play a key hierarchical role in cardiac pathophysiology via glycan-dependent regulation of inflammatory responses. Here, we discuss the emerging roles of Gal-1 in cardiovascular diseases including acute myocardial infarction, heart failure, Chagas cardiomyopathy, pulmonary hypertension, and ischemic stroke, highlighting underlying anti-inflammatory mechanisms and therapeutic opportunities. Whereas Gal-1 administration emerges as a potential novel treatment option in acute myocardial infarction and ischemic stroke, Gal-1 blockade may contribute to attenuate pulmonary arterial hypertension.

## 1. Introduction

Galectins are a family of *β*-galactoside-binding lectins widely expressed in a variety of cells and tissues [1]. These animal lectins play critical roles in the regulation of both innate and adaptive immunity, inflammation, wound healing, and angiogenesis [2–6]. To date, 15 different members have been identified, which are classified according to their biochemical structure into three groups: (a) prototype galectins, including galectin- (Gal-) 1, -2, -5, -7, 10, -11, -13, -14, and -15, display one carbohydrate-recognition domain (CRD) and can dimerize; (b) tandem-repeat galectins, including Gal-4, -6, -8, -9, and -12, exhibit two CRDs associated with a linker peptide; and (c) the unique chimera-type Gal-3 contains one CRD and an additional nonlectin domain and can oligomerize [1]. Emerging evidence demonstrates that galectins play important roles in cardiovascular pathophysiology by controlling acute and chronic inflammatory responses [7]. Despite structural similarities, individual members of the galectin family play distinct roles during inflammatory processes by controlling innate and adaptive immune compartments [8]. This includes modulation of proinflammatory cytokine production, promotion of T regulatory (Treg) cell expansion, and control of immune cell differentiation and survival [9].

### 1.1. Galectin-1 (Gal-1)

Gal-1, the first galectin identified, functions mostly as an anti-inflammatory mediator that represses innate and adaptive immune programs [1, 9]. Expression of Gal-1 is prominent in inflammatory macrophages [10, 11], activated T lymphocytes [12], Treg cells [13], and tolerogenic dendritic cells [14]. From a structural viewpoint, Gal-1 consists of two subunits of 14.5 kDa (135 amino acids) present in a dynamic dimerization equilibrium [15]. Because of an unusual number of six cysteine residues, this lectin is highly susceptible to oxidative inactivation, which hampers its immunoregulatory activity [16]. Gal-1 binds to multiple galactose-*β*1-4-N-acetyl-glucosamine (N-acetyl-lactosamine; LacNAc) units present on the branches of N- or O-linked glycans on a variety of cell surface receptors and modulates their segregation, endocytosis, and signaling [4, 15]. These glycan structures are generated by the coordinated action of a set of glycosyltransferases including N-acetylglucosaminyltransferase 5 (MGAT5), an enzyme that creates *β*1,6-N-acetylglucosamine-branched complex N-glycans, and core-2 *β*1-6-N-acetylglucosaminyltransferase 1 (C2GNT1), an enzyme that catalyzes branching of core-2 O-glycan structures. Conversely, Gal-1 binding is prevented when LacNAc is modified by *α*2,6-linked sialic acid incorporated by *α*2,6-sialyltransferase 1 (ST6GAL1) [3]. Gal-1 controls biological processes through extracellular mechanisms by cross-linking cell surface glycoconjugates or intracellularly by influencing a variety of signaling events [1, 2]. Through glycosylation-dependent mechanisms, this lectin modulates cellular activation, proliferation, migration, and survival, playing a critical role in the resolution of acute and chronic inflammation [4–6, 15]. In addition, Gal-1 promotes neovascularization by signaling through vascular endothelial growth factor receptor- (VEGFR-) 2 [17, 18]. Because of its prominent anti-inflammatory, proresolving, and proangiogenic activities, Gal-1 has been proposed to be a key mediator of cardiovascular homeostasis [19].

Here, we discuss the relevance of Gal-1 in cardiovascular disorders including acute myocardial infarction (AMI), heart failure (HF), Chagas cardiomyopathy, pulmonary hypertension (PAH), and ischemic stroke and highlight cellular and molecular mechanisms underlying these effects.

### 1.2. Acute Myocardial Infarction

Acute myocardial infarction (AMI) is caused by a sudden interruption in blood oxygen supply mostly due to atherothrombosis; this effect is accompanied by a storm of proinflammatory cytokines and a dysregulation of effector and regulatory immune cell mechanisms [20]. AMI is the leading cause of death worldwide, and those who survive are at higher risk for adverse ventricular remodeling and heart failure [21].

**Figure 1:**
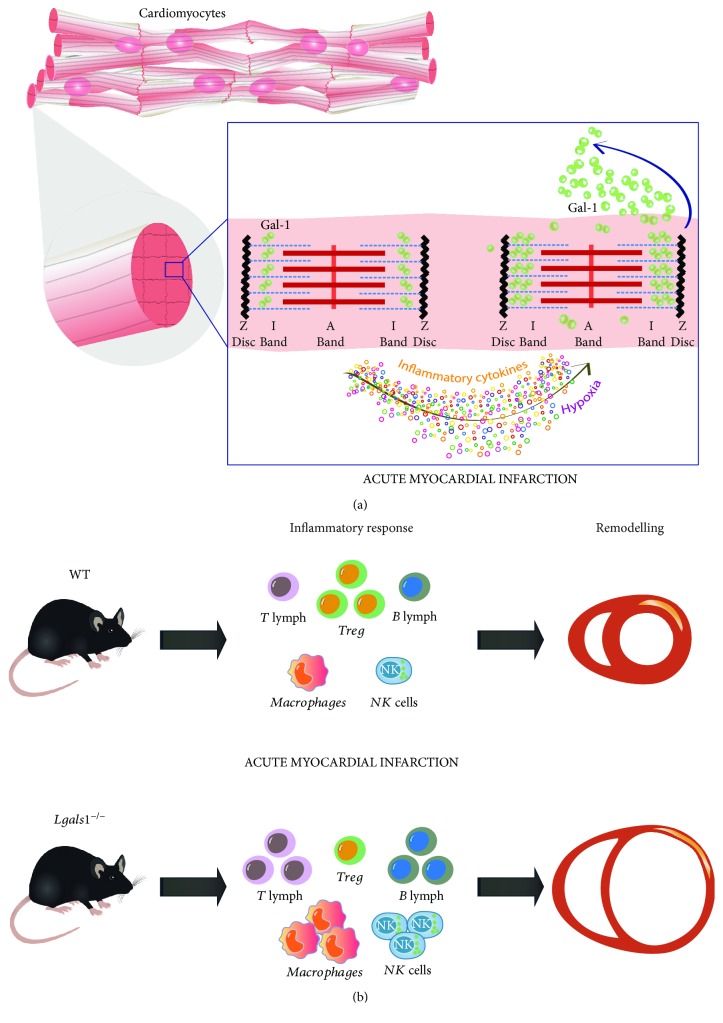
Role of Gal-1 in acute myocardial infarction. (a) Gal-1 is constitutively expressed in cardiomyocytes close to sarcomeric actin and is increased and secreted after acute myocardial infarction (AMI), inflammation, and hypoxia. (b) Mice lacking Gal-1 (*Lgals1^−/−^*) show adverse ventricular remodeling after AMI associated with increased inflammation and reduced proportion of regulatory T (Treg) cells.

Gal-1 is expressed in the normal heart of many species [22–24]. Cardiomyocytes constitutively express Gal-1 at the cytosolic compartment in an organized striated pattern close to sarcomeric actin but not myosin [23] (Figure 1(a)). However, Gal-1 expression is further upregulated during AMI, with a peak as early as 20 min after experimental coronary artery ligation [24], followed by a second peak at 7 days [19]. This bimodal expression pattern may reflect different cells that express Gal-1 in AMI either ischemic cardiomyocytes (20 min) or leukocytes that promote resolution of inflammation (7 days).

Cultured cardiomyocytes upregulate and secrete Gal-1 early after hypoxia and in response to proinflammatory cytokines [19], as seen in the early phase of AMI *in vivo* (Figure 1(a)). Whereas early upregulation of Gal-1 may serve as a homeostatic safeguard mechanism to prevent a dysregulated inflammatory response that could otherwise promote adverse remodeling [20], augmented Gal-1 expression later in the course of AMI could influence the resolution of cardiac inflammation and restore homeostasis [25].

Interestingly, Gal-1 was found to be upregulated 7 days after nonreperfused AMI, associated with a peak in infiltrating dendritic cells, lymphocytes, and macrophages [26]. Since Gal-1 promotes differentiation of tolerogenic dendritic cells and M2-type macrophages [14, 27] which play protective roles in AMI [28, 29], Gal-1-glycan interactions may contribute to reparative functions mediated by these cells.

Within the T-cell compartment, Gal-1 promotes apoptosis of CD8 and Th1 and Th17 lymphocytes, promoting a shift toward a Th2-dominant cytokine profile [3, 30, 31]. This effect is highly relevant in cardiovascular diseases as upregulation of Th1 and Th17 cytokines is a typical hallmark observed in patients with acute coronary syndromes [32–34] and is associated with adverse remodeling after AMI [32]. On the other hand, healthy patients exhibiting Th2-dominant responses may be protected from cardiovascular disease [35]. Thus, Gal-1 emerges as an attractive therapeutic candidate to limit innate and adaptive responses early or late during cardiovascular inflammation. Remarkably, mice lacking Gal-1 (*Lgals1^−/−^*) showed adverse ventricular remodeling after AMI with increased cardiac dilation associated with dysregulated uncontrolled inflammation (Table 1) [19]. Absence of Gal-1 led to an increase in cardiac infiltration by T lymphocytes, macrophages, and natural killer (NK) cells, while anti-inflammatory Treg cells were significantly reduced in this setting (Figure 1(b)) [19]. As Treg cells are protective in AMI [36], and Gal-1 is also a critical mediator of Treg function [13, 37], Gal-1-driven inhibitory circuits may also operate in Treg-mediated protection during AMI.

As mentioned above, Gal-1 is expressed at the cytosolic compartment of cardiomyocytes in association with actin and is secreted in response to injury. Since *Lgals1^−/−^* mice lack both the intracellular and extracellular Gal-1 activities, it is difficult to infer which regulatory function of this lectin controls adverse remodeling after AMI. However, treatment with recombinant Gal-1 has been used to address the extracellular versus intracellular roles of this lectin. Mice treated with a single dose of recombinant Gal-1 during AMI showed a significant improvement in ventricular function and remodeling (Table 2) [19]. These effects support the concept that extracellular activities of Gal-1 prevail in cardioprotection and highlight the therapeutic potential of this lectin in patients with AMI. Interestingly, exogenous Gal-1 also prevented renal ischemia-reperfusion injury through anti-inflammatory mechanisms [38]. Nevertheless, as Gal-1 can be taken up by cells devoid of this lectin [39], this alternative mechanism could also operate in cardiomyocytes. Thus, in addition to the anti-inflammatory effects of the exogenous protein, Gal-1-driven nonimmunological events may also take place.

Atherosclerosis is the underlying pathology for most AMIs. Atherosclerotic plaque rupture leads to coronary thrombosis and myocardial necrosis. Inflammation plays a key role in both development and fate of atherosclerosis [40]. A recent large clinical study showed that inhibition of atherosclerosis improved clinical outcomes in patients with previous AMI [41]. Experimental atherosclerosis in *ApoE^−/−^* mice fed with cholesterol showed increased Gal-1 expression in atherosclerotic plaques both in the media and in the intima layer [42]. However, in broad contrast to Gal-3, Gal-1 expression was not increased over time. Moreover, statin treatment led to inhibition of Gal-3 but had no effect on Gal-1 expression [42]. Although the role of Gal-1 in atherosclerosis has not yet been examined in detail, Gal-3 blockade led to reduced atherosclerosis in *ApoE^−/−^* mice [43, 44].

### 1.3. Heart Failure

Patients who experience adverse ventricular remodeling after AMI are at increased risk of developing heart failure (HF). Although mortality after AMI decreased over the last decades, HF experienced a significant increase [21]. HF may also result from dilated nonischemic cardiomyopathy in the absence of AMI. The worldwide burden of HF is increasing, representing a global health problem. Despite advances in both pharmacological approaches and left ventricle assisting devices, the only available treatment for patients with end-stage HF is cardiac transplantation. Given the role of Gal-1 as an important regulator of immune responses, Gal-1 expression was investigated in patients with advanced HF. Heart samples explanted from HF patients undergoing transplantation showed increased Gal-1 expression compared to control hearts [19]. As expected, Gal-1 was localized within the inflammatory infiltrate and the interstitium, but was also found in cardiomyocytes. Accordingly, Gal-1 was upregulated in hearts from Chagas cardiomyopathy patients [45]. Whether Gal-1 expression in the heart of HF patients is part of the pathogenic mechanisms of the disease or may represent a compensatory effect in response to enhanced inflammation is still not clear. Further studies in patients with ischemic as well as nonischemic HF are warranted to better understand the role of Gal-1 in both etiology and prognosis of this disease. Moreover, as different environmental factors may influence Gal-1 expression including hypoxia, inflammation, aging, and metabolic status [3, 17], further work is needed to dissect the role of these factors in regulating the activity of this lectin.

Interestingly, mice lacking Gal-1 showed mild ventricular dilation, reduced contractility, and an enhanced inflammatory infiltrate composed of lymphocytes, macrophages, and NK cells, as well as reduced number of Treg cells, indicative of autoimmune myocarditis (Table 1) [19]. Moreover, *Lgals1^−/−^* mice showed increased levels of circulating Th1 and Th17 cytokines [3] which might contribute to ventricular dysfunction and dilation, similar to the dysfunction observed in septic patients [46], as well as experimental inflammation induced by interleukin- (IL-) 1*β* and IL-18 [47, 48]. The upregulated expression of Gal-1 in patients with HF may therefore represent a homeostatic mechanism that controls autoimmune myocarditis in response to injury. Whether intracellular Gal-1 participates in cardiomyocyte contraction and may contribute to cardiomyopathy and HF is still uncertain.

### 1.4. Chagas Disease

Chagas disease is caused by infection with the protozoan parasite *Trypanosoma cruzi* (*T. cruzi*). Acute infection is usually mild and undiagnosed; however, 20–30% of patients develop dilated cardiomyopathy several years later [49]. Although the precise mechanisms underlying Chagas cardiomyopathy are not completely understood, they involve (a) injury to cardiomyocytes induced by *T. cruzi*, (b) cardiac inflammation, (c) microvascular dysfunction, and (d) autonomic disorders [50].

The *T. cruzi* parasite itself does not express Gal-1, but anti-Gal-1 antibodies have been observed in patients with Chagas disease: whereas IgM and IgE antibodies raise with acute infection, anti-Gal-1 IgG antibodies are present in the chronic phase of the disease [45]. The source of this humoral response was mainly associated to antigen release from host cells, since no cross-reactivity was found between Gal-1 and *T cruzi* proteins. Likewise, autoantibodies against other host cell proteins have been observed in Chagas disease patients [51]. In fact, serum Gal-1 increases in patients with Chagas disease irrespective of cardiac involvement [52].

Infection with *T. cruzi* induces the release of interferon-*γ* (IFN-*γ*), which promotes death of intracellular parasites, thus limiting the extent of infection [50]. However, IFN-*γ* and uncontrolled inflammation may also lead to host damage and myocarditis [53]. Thus, regulation of Gal-1 expression by IFN-*γ* [19] may contribute as a homeostatic mechanism to counterbalance cardiac inflammation. Whereas a study found that Gal-1 binds weekly to *T. cruzi* with no direct effect [54], another study showed no significant binding to the parasite [52]. On the contrary, Gal-1 appears to be upregulated after infection and contributes to parasite-immune escape. Activated B lymphocytes from infected mice secreted high levels of Gal-1 which promoted apoptosis of activated T lymphocytes [55]. Moreover, infected dendritic cells also secreted Gal-1 which contributed to expansion of the Treg cell compartment, leading to inhibition of CD8^+^ T-cells. These results suggest that Gal-1 may limit the clearance of *T. cruzi* parasite by triggering tolerogenic circuits [54]. However, the direct effect of *T. cruzi* on cardiomyocytes involves different mechanisms. Whereas *T cruzi* infects cardiomyocytes and induces apoptosis [56], infected cardiomyocytes may secrete Gal-1 as a cardioprotective mechanism, as Gal-1 affinity to cardiomyocytes prevents *T. cruzi* infection via glycosylation-dependent mechanisms [52]. Whether this pathway involves inhibition of *T. cruzi* binding to cardiomyocytes through hindrance of glycan-binding sites remains uncertain. Interestingly, infected cardiomyocytes show an altered glycophenotype which prevents Gal-1 binding, promoting parasite escape [52]. Thus, Gal-1 may control the fate and function of cardiomyocytes through direct or indirect mechanisms involving immune-dependent or independent pathways.

Despite the immunosuppressive effects of Gal-1, studies in *T. cruzi*-infected *Lgals1^−/−^* mice showed reduced cardiac inflammation [52] and lower myocardial parasite infiltration (Table 1) [54]. Results on survival and parasitemia in those mice were however controversial: as one study showed longer survival despite increased parasitemia [52], another study indicated reduced survival with similar parasitemia [54]. Differences in these parameters may depend on the *T. cruzi* strain: *in vitro* cardiomyocyte infection with Brazil, but not Tulahuen strain of *T. cruzi*, showed an early mild reduction of Gal-1 expression [52]. In this regard, *in vivo* infection with *T. cruzi* Y strain showed higher cardiac inflammation and mortality than the VFRA and Sc43 strains [57]. Thus, different parasite strains may differentially affect Gal-1 expression, selective binding to glycosylated receptors on host cells, and clinical outcome of *in vivo* infection. Of note, all studies were performed in *T. cruzi-*infected mice and may not fully recapitulate the pathophysiology of Chagas cardiomyopathy, which takes place several years after infection and may not be associated with parasitemia or myocardial parasite infiltration. Finally, survival of these septic mice should not be considered as a surrogate for Chagas cardiomyopathy. Unfortunately, no animal models have completely recapitulated the pathophysiology and clinical course of human Chagas cardiomyopathy.

### 1.5. Pulmonary Arterial Hypertension

Pulmonary arterial hypertension (PAH) is defined as a sustained increase in mean pulmonary artery pressure in the absence of an elevation in pulmonary artery wedge pressure [58]. Symptoms consist of dyspnea and right ventricle (RV) overload leading to heart failure and death. The hallmark of PAH is proliferation of vascular smooth muscle cells (VSMCs) that cause arterial narrowing leading to an increase in vascular resistance [59]. However, endothelial cells (ECs) control VSMC function, contraction, and proliferation through soluble mediators, direct contact, and extracellular vesicles [60].

Gal-1 is expressed and secreted by both ECs and VSMCs. This lectin modulates VSMC attachment, spreading, and migration through affinity to extracellular matrix glycoproteins including laminin, fibronectin, and *α*_1_*β*_1_ integrin [61]. Both vascular deposits of laminin and fibronectin are typically observed in PAH [59, 62]. Interestingly, the precise effects of Gal-1 on VSMC are controversial: whereas some studies showed inhibition of spreading [60] and migration [63], one study showed increased DNA replication following exposure to this lectin [64].

Chronic hypobaric hypoxia, an established model of PAH, leads to upregulation of lung Gal-1 expression [65]. Mice lacking Gal-1 showed reduced PAH in this model with ameliorated RV hypertrophy (Table 1) [65]. Despite the *in vitro* effects of Gal-1 on VSMCs, arterial muscularization and microvessel density were not affected in Gal-1-deficient mice. However, absence of Gal-1 in the lungs was associated with decreased vasoreactivity after acute hypoxia, with no effects in vasopressor response after 5-hydroxytryptamine and potassium chloride [65]. Interestingly, effects on vascular reactivity could be associated to calcium influx in VSMCs, since intracellular Gal-1 has been shown to inhibit ICa_,L_ modifying the Ca_v_1.2 calcium channel in a splice variant-dependent manner [66]. In this regard, Gal-1 promotes Ca_v_1.2 degradation in smooth muscle cells controlling vascular reactivity and blood pressure [67]. Moreover, Gal-1 has also been implicated in the pathogenesis of preeclampsia, which is characterized by hypertension and proteinuria during pregnancy [68], suggesting new roles for this lectin in pulmonary, systemic, and pregnancy-induced arterial hypertension.

Remarkably, PAH increases RV afterload leading to contractile dysfunction and Gal-1 expression in the normal heart has been shown to be more pronounced in RV [24]. The contribution of cardiomyopathy to the pathogenesis of PAH in Gal-1-deficient mice warrants further consideration. Moreover, the phenotype of *Lgals1^−/−^* PAH mice appears to be the combination of the absence of this lectin in the lung vasculature, the RV, and the contribution of the immune system. Further experiments are warranted to investigate the translational and therapeutic implications of these findings.

### 1.6. Stroke

Similar to AMI, stroke is characterized by a sudden drop in oxygen blood supply to the brain. In the normal brain, Gal-1 is expressed by both neurons and glia [69, 70] and modulate astrocyte function [71]. Gal-1 upregulation is observed *in vitro* after anoxia [71] and *in vivo* in the infarcted and penumbra area after experimental stroke [72]. Gal-1 has been shown to induce astrocyte differentiation and brain-derived neurotrophic factor (BDNF) secretion [71]. However, Gal-1 treatment inhibited normal astrocyte proliferation *in vitro* [73] and promoted microglial deactivation [27]. *In vivo*, Gal-1 treatment promoted neurogenesis after stroke in a carbohydrate-dependent manner, and antibody-mediated Gal-1 blockade prevented this effect (Table 2) [72]. Similar to Gal-1 treatment, transfer of stem cells overexpressing Gal-1 significantly reduced infarct volume [74]. Stroke confers an oxidative environment which inactivates Gal-1 impairing some carbohydrate-binding activities. However, oxidized Gal-1 stimulates axonal regeneration [75]. Besides its role in neuronal proliferation after injury, Gal-1 showed a neuroprotective effect through inhibition of central nervous system (CNS) inflammation. Interestingly, Gal-1 treatment induced an M2 microglia phenotype, which prevented inflammation-induced neurodegeneration [27]. On the contrary, an M1 “inflammatory” phenotype was associated with CNS toxicity [76]. Gal-1 binds with higher affinity to M1 microglia skewing the balance toward an M2 phenotype [27]. In addition, Gal-1 modifies the glutamate receptor NMDA preventing binding of this neurotransmiter [77]. Furthermore, this lectin participates in neuronal development, since *Lgals1^−/−^* mice display altered olfactory neurons [78] and reduced thermal sensitivity [79].

Serum Gal-1 levels have been evaluated in patients with stroke. Whereas one study showed normal values of this protein [80], a larger cohort showed increased serum Gal-1 after ischemic stroke [81]. Both studies, however, found no association between Gal-1 levels and functional recovery. Thus, serum Gal-1 does not appear to be a reliable biomarker of local activation or neuroprotection.

### 1.7. Other Galectins

In addition to Gal-1, other members of the galectin family may control cardiovascular pathophysiology. Although their functions are beyond the scope of the present review, we will briefly summarize their most important contributions to cardiovascular diseases. Remarkably, Gal-3 has been widely studied in the context of cardiovascular disease and its effects and potential diagnostic and prognostic values have been extensively reviewed elsewhere [7, 8]. This “chimera-type” lectin is highly expressed by macrophages, promoting inflammation, and fibrosis in various organs [82]. In AMI, Gal-3 is upregulated early and released to the extracellular compartment. Mice lacking Gal-3 (*Lgals3^−/−^*) showed improved cardiac function and reduced fibrosis after pressure overload and during hypertension [83]. On the contrary, adverse remodeling with increased infarct size and reduced scar fibrosis was observed in *Lgals3^−/−^* mice after AMI, probably due to ventricular rupture [84]. Gal-3 promotes cardiac as well as renal, hepatic, vascular, and pulmonary fibrosis [8, 85]. Several clinical studies reported increased serum levels of Gal-3 in cardiovascular diseases, and this lectin is currently available as a cardiovascular risk biomarker for heart failure [86]. Moreover, Gal-3 has been proposed as a biomarker that links oxidative stress and inflammation in patients with atherothrombosis [87]. In addition, Gal-3 levels are also increased in patients with AMI although the clinical value of this lectin is still a matter of debate [8].

Although less studied, Gal-2 has also been shown to play an important role in cardiovascular disease. Similar to Gal-1, Gal-2 is a prototype lectin that promotes apoptosis of activated CD8^+^ T cells [88, 89]. However, in contrast to Gal-1, Gal-2 also showed proinflammatory activity within the monocyte/macrophage compartment promoting a shift towards an M1 phenotype [90] and inhibiting arteriogenesis [91]. Interestingly, Gal-2 is elevated in atherosclerotic plaques and colocalized with lymphotoxin-*α* [91, 92], suggesting a galectin-cytokine interaction that favors proinflammmatory responses. Whereas a study in the Japanese population showed a functional single-nucleotide polymorphism (SNP) rs7291467 (C3279C➔T) in the Gal-2 gene (*LGALS2*) that was associated with increased risk of AMI [92], these results could not be confirmed in Caucasian individuals, and a meta-analysis of seven studies showed no association between *LGALS2-C3279T* and coronary artery disease [93].

Gal-9, a tandem-repeat member of the galectin family, functions mostly as an anti-inflammatory lectin [94] that promotes Th1 and Th17 apoptosis through interaction with T-cell immunoglobulin domain and mucin domain protein 3 (TIM-3) [2, 4]. Gal-9 has shown to prevent renal ischemia/reperfusion injury, to attenuate experimental viral myocarditis [95], and to improve survival after cardiac transplantation [96]. Further studies are needed to unravel the precise roles of Gal-9 in AMI and other cardiovascular diseases.

Finally, Gal-12, a tandem-repeat galectin preferentially expressed in adipose tissue, contributes to differentiation and turnover of preadipocytes [4, 97]. Targeted disruption of Gal-12 gene (*Lgals12*) in mice increased insulin sensitivity and glucose tolerance and contributed to diabetes and metabolic syndrome [4, 97], suggesting the potential role of this lectin in cardiovascular diseases.

Although a preferential set of receptors have been described for individual members of the galectin family [98], some glycosylated receptors may be shared by some galectins. This includes CD45 which may be engaged by both Gal-1 and Gal-3 on T lymphocytes [99] and VEGFR2 which mediates the function of those galectins on ECs [18, 100]. Since different galectins may induce antagonic effects (e.g., proinflammatory versus anti-inflammatory effects), potential cross-talk between these glycan-binding proteins and competition for similar receptors or glycan structures should also be taken into consideration.

## 2. Conclusions

**Figure 2:**
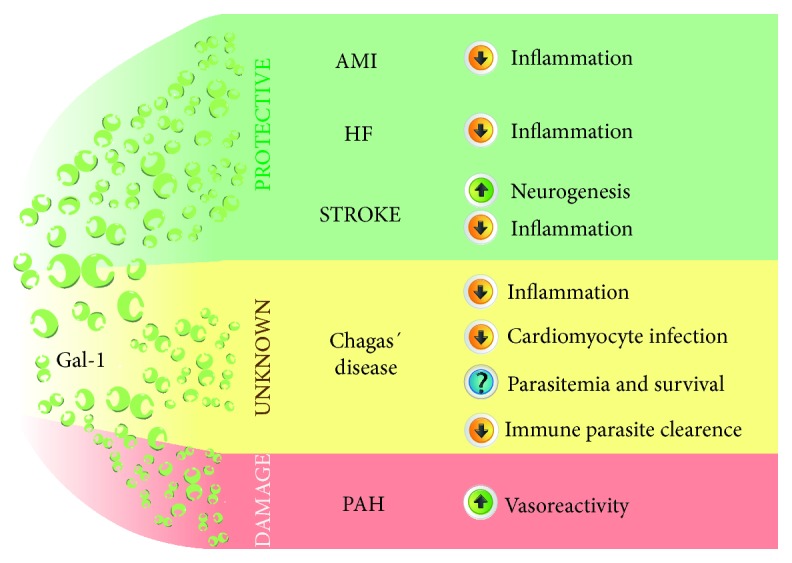
Proposed roles of Gal-1 in cardiovascular disease. Galectin-1 (Gal-1) has been implicated in a number of cardiovascular disorders. The roles of this lectin vary according to different clinical contexts. Gal-1 = galectin-1; AMI = acute myocardial infarction; HF = heart failure; PAH = pulmonary arterial hypertension.

Galectins, a family of *β*-galactoside-binding lectins, have emerged as key modulators of inflammatory processes [1–4]. Gal-1, a highly conserved member of this family, is widely expressed in different tissues and contributes to immunosuppression in cancer, infection, and autoimmune diseases [1–4]. Recently, an emerging role for Gal-1 in cardiovascular processes has been proposed (Figure 2). Gal-1 is constitutively expressed in cardiomyocytes complexed with cytosolic actin. Interestingly, Gal-1 expression is increased in the heart of patients with AMI, HF, and Chagas cardiomyopathy. Mice lacking Gal-1 show increased susceptibility to chronic inflammatory diseases and develop autoimmune myocarditis and cardiac dysfunction, suggesting its central nonredundant roles in cardiac homeostasis. Through binding to cell surface ligands, Gal-1 not only controls innate and adaptive immune programs that lead to resolution of inflammatory responses, but also may control cardiomyocyte survival leading to potential applications of this lectin in the treatment of AMI and other cardiovascular disorders. Accordingly, Gal-1 has been associated to the pathogenesis of PAH and ischemic stroke (Figure 2). Further studies should be aimed at defining specific roles of Gal-1 in the heart, identifying its candidate receptors within cardiac tissue and unveiling potential interplay with other members of the galectin family, with the ultimate goal of designing and implementing galectin-based therapies. Several strategies have been proposed to modulate Gal-1 expression and function, including anti-Gal-1 antibodies in cancer and infection settings [17, 101] as well as recombinant Gal-1 in autoimmune and chronic inflammatory disorders [3, 31]. From a translational standpoint, recombinant Gal-1 administration emerges as the most attractive approach for treatment of cardiovascular diseases, particularly AMI [19]. On the other hand, as Gal-1 cross-links and signals via glycosylated receptors, an alternative strategy could potentially involve modulation of glycans generated by the concerted action of glycosyltransferases, including MGAT5, C2GNT1, and ST6GAL1 [1]. Although mice lacking these glycosyltransferases display clear immunological phenotypes, there is still no information on their role in cardiovascular inflammation. Moreover, no studies have been performed to evaluate the impact of glycosyltransferase inhibitors in these pathologies. Although distinct Gal-1 receptors have been identified on the surface of different cell types, including CD45 and CD43 on T cells [99], CD45 on microglia [27], and VEGFR2 on ECs [18], its potential counter-receptors and glycosylated ligands on the surface of cardiomyocytes have not yet been explored. Finally, further studies should be conducted to validate the above mentioned mechanisms in clinical settings. Although animal models represent an invaluable tool for understanding cardiovascular pathology, clinical studies are essential to definitely validate galectin-driven circuits as patients may exhibit other pathologic conditions which could also influence Gal-1 expression and function.

## Figures and Tables

**Table 1:** Preclinical models of cardiovascular disease in *Lgals1^−/−^* mice.

Model	Effects	Mechanisms	Reference
AMI	Worse remodeling	Increased inflammation	[19]
HF	Spontaneous CMP	Increased inflammation	[19]
Chagas disease	Reduced cardiac inflammation and parasite infection	Not fully understood. Controversial results on survival	[52, 54]
PAH	Less PAH and reduced RV hypertrophy	Decreased vasoreactivity	[65]

AMI = acute myocardial infarction; HF = heart failure; PAH = pulmonary arterial hypertension; CMP = cardiomyopathy; RV = right ventricle.

**Table 2:** Effects of Gal-1 treatment on cardiovascular disease.

Model	Effects	Mechanisms	Reference
AMI (reperfused)	Improved remodeling	Inflammation? (not evaluated)	[19]
Stroke	Improved sensorimotor dysfunction	Increased neurogenesis. No effect on infarct volume	[72]

AMI = acute myocardial infarction; HF = heart failure; PAH = pulmonary arterial hypertension.
